# Variation in thyroid volumes due to differences in the measured length or area of the cross‐sectional plane: A validation study of the ellipsoid approximation method using CT images

**DOI:** 10.1002/acm2.13125

**Published:** 2021-03-29

**Authors:** Naotoshi Fujita, Katsuhiko Kato, Shinji Abe, Shinji Naganawa

**Affiliations:** ^1^ Department of Radiological Technology Nagoya University Hospital Nagoya Japan; ^2^ Department of Radiological and Medical Laboratory Sciences Nagoya University Graduate School of Medicine Nagoya Japan; ^3^ Functional Medical Imaging Biomedical Imaging Sciences Division of Advanced Information Health Sciences Department of Integrated Health Sciences Nagoya University Graduate School of Medicine Nagoya Japan; ^4^ Department of Radiology Nagoya University Graduate School of Medicine Nagoya Japan

**Keywords:** CT, ellipsoid approximation, graves' disease, internal radioiodine therapy, thyroid, volumetry

## Abstract

**Purpose:**

This study examined the variation in the thyroid volume determined by the ellipsoid approximation method due to differences in the measured length or area of the cross‐sectional plane of CT images.

**Methods:**

Forty‐five patients with Graves' disease were included in this retrospective study. We designated the three‐dimensional thyroid volumes extracted manually (*V*_CT_) as the reference data and calculated five approximate volumes for comparison: (a) the mean volume of 8100 different thyroid volumes depending on the diameter of the cross‐sectional plane at the midpoint of the major axis, (*V*_ellipsoid,mean_); (b) the volume using the maximum diameter and its orthogonal diameter, (*V*_ellipsoid,maxlength_); (c) the maximum (*V*_ellipsoid,maxvolume_); (d) minimum (*V*_ellipsoid,minvolume_) of the 8100 thyroid volumes; and (e) the volume determined with an equivalent circle diameter, (*V*_ellipsoid,Heywood_).

**Results:**

Thyroid volumes obtained via the ellipsoid approximation method varied depending on the diameter of the cross‐sectional plane and included a mean error of approximately 20%, while the concordance correlation coefficient (CCC) differed for each approximate volume. Among these volumes, *V*_ellipsoid,mean_ and *V*_ellipsoid,Heywood_ were in good agreement with *V*_CT_, according to single regression analyses and the resultant CCC values, with mean errors of 0.1% and 10.4%, respectively.

**Conclusion:**

While *V*_ellipsoid,Heywood_ approximated thyroid volumes with vastly reduced errors, we recommend utilizing three‐dimensional thyroid volumetry if measurement accuracy is required.

## 1 Introduction

Internal radioiodine therapy (iodine‐131) is a treatment for Graves' disease.^1^ In radioiodine therapy for Graves' disease, the administered radioactivity is often determined based on the patient's thyroid volume.^2^, ^3^, ^4^ Therefore, accurate thyroid volumetry before radioiodine therapy leads to the accurate determination of the administered radioactivity, thereby ensuring the therapeutic effect. Thyroid volumetry involves analyses of images obtained from: (a) scintigraphy,^5^, ^6^ (b) ultrasonography,^7^ and (c) computed tomography (CT).^8^, ^9^, ^10^ Utilizing these images, the thyroid is approximated as a complex of ellipsoids, known as the ellipsoid approximation method, and it has been in popular use owing to its inherent simplicity. In the ellipsoid approximation method, it is customary to use the maximum diameter of the cross‐sectional plane (transverse plane of the body), although there is no clear rule in either past papers or guidelines. Schlögl et al.^11^ compared the accuracy of the thyroid volume determined by the ellipsoid approximation method and the three‐dimensional segmentation of ultrasound images. In their study, the ellipsoid volumes were determined by measuring the maximum transversal, horizontal, and longitudinal diameters. They reported that the approximate volume was 11% larger than the actual volume with a standard deviation of 26%. However, they did not report on the reason for using the maximum diameter, the method used to measure each diameter, and its variation. We believe the differences in the measured length or area of the cross‐sectional plane particularly affect the ellipsoid and thus the approximated thyroid volume. Furthermore, the accuracy of the measurements depends on the subjectivity and skill of the measurers (i.e., physicians, radiological technologists, etc.). The results of three‐dimensional thyroid volumetry from CT images were reported to be in good agreement with the actual thyroid volume by Lee et al.^10^ They targeted patients who underwent contrast‐enhanced CT examination, and the thyroid regions were extracted from the CT images using a three‐dimensional visualization software. However, contrast‐enhanced CT scans will delay radioiodine therapy for weeks or months because the contrast media contains iodine.^3^, ^4^ Therefore, direct application of their approach is difficult for patients with Graves' disease before radioiodine therapy.

Regardless of the modality employed, it is significant to validate an accurate and simple method for measuring the thyroid volume. We need to investigate how the thyroid volume changes with various diameter combinations, including the combination of the maximum and orthogonal diameter of the cross‐sectional plane. This study aimed to calculate the variation in the thyroid volume determined by the ellipsoid approximation method due to differences in the measured length or area of the cross‐sectional plane of CT images.

## 2 Materials and Methods

### 2.1 Patients

Forty‐five patients (7 males, 38 females, mean patient age of 50.8 ± 16 years) with Graves' disease who underwent radioiodine therapy as outpatients from December 2014 to April 2018 were included in this retrospective study, approved by the Ethical Review Committee of Nagoya University Hospital (authorization no. 2018‐0179). Because five of this cohort underwent radioiodine therapy twice during this period, our analysis eventually included 50 cases. In our hospital, we determined the administered radioactivity of radioiodine for Graves' disease according to Marinelli's formula.^2^ Using Marinelli's formula (Eq. 1), the administered radioactivity, *A*, was calculated using the thyroid volume, among other parameters. For this reason, patients underwent non‐enhanced neck CT examinations (Aquilion 64 or Aquilion PRIME SP; Canon Medical Systems, Otawara, Japan) for the determination of thyroid volumes. The scan parameters were as follows: X‐ray tube voltage, 120 kVp; X‐ray tube current, use automatic exposure control (preset noise index (standard deviation, SD), 8 or 11); mean CT dose index (CTDI_vol_), 13.0 ± 5.4 mGy; rotation speed, 0.5 s/rotation; field of view, 200 mm; in‐plane resolution, 0.39 mm/pixel; slice thickness/gap, 5 mm/5 mm; reconstruction kernel, a standard soft‐tissue kernel (FC13 as Aquilion 64, FC03 as Aquilion PRIME SP). Under this scan condition, the in‐plane spatial resolutions of both systems are comparable. In this study, we performed retrospective analyses using these neck CT images.

The Nuclear Medicine physician determines the administered radioactivity by adjusting the absorbed dose in Equation 1 according to the clinical symptoms of each patient.

(1) $$\begin{array}{c} \begin{array}{c} A\left[\mathrm{MBq}\right]=C\times \frac{D\times V}{U\times T_{\text{eff}}} \end{array} \end{array}$$

where *A* is the administered radioactivity (MBq), *D* is the thyroid absorbed dose (Gy), *V* is the thyroid volume (mL), *U* is the radioiodine uptake fraction at 24 hrs after administration, *T*_eff_ is the effective iodine‐131 half‐life (d), and *C* is the unit conversion coefficient (MBq d Gy^‐1^ g^‐1^). In our hospital, we substitute 0.185 for *C*. Hereafter, the density of the thyroid is assumed to be 1 g/ml.

### 2.2 Three‐dimensional thyroid volumetry using CT images

**Fig. 1:**
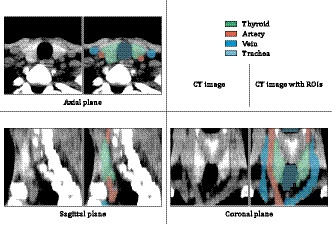
An example of segmentation processing using a 3D slicer. The thyroid region (green) was extracted while excluding the surrounding artery (red), vein (blue), trachea (cyan), or muscles.

According to Shu et al.^9^ and Lee et al.,^10^ the results of three‐dimensional thyroid volumetry using CT images were in g.od agreement with the actual thyroid volume. Schlögl et al.^11^ also reported similar results using ultrasound images. Therefore, we designated the three‐dimensional thyroid volumes extracted manually as the reference data (*V*_CT_) in this study. First, the thyroid region of interest (ROI) in the CT images was manually extracted from the CT images with a slice thickness of 5 mm (3D Slicer software version 4.10.1). Figure 1 shows an example of segmentation processing using the 3D Slicer. The thyroid region was extracted while excluding the surrounding blood vessels or muscles. Then, the sum of the voxels in each slice was calculated as the thyroid volume (*V*_CTpre_). This study was a retrospective analysis and we could not verify the data using thin slice CT images. Therefore, referring to the method of Veres et al.,^12^ we acquired the CT images with 0.5‐ to 10‐mm slices for a sphere (38‐mm diameter, 28.7 cm^3^) with a known volume (true volume, *V*_true_), and preliminarily verified the variance in volume owing to slice thickness. The volume obtained from each slice thickness (measured volume, *V*_measured_) was measured three times by the same operator. Then we obtained the correction factor (*W*_PVC_) for each slice thickness, *T* (mm) by dividing the *V*_measured_ by the *V*_true_ (Eq. 2) and linear regression equation between *T* and *W*_PVC_. Finally, the partial volume‐corrected thyroid volume (*V*_CT_) is obtained by Eq. 3.

(2) $$\begin{array}{c} \begin{array}{c} W_{\text{PVC}}=\frac{V_{\text{measured}}}{V_{\text{true}}} \end{array} \end{array}$$

(3) $$\begin{array}{c} \begin{array}{c} V_{\text{CT}}=\frac{V_{\text{CTpre}}}{W_{\text{PVC}}} \end{array} \end{array}$$

### 2.3 Hyroid volumetry using ellipsoid approximation

The ellipsoid approximation method for thyroid volumetry was described by Malago et al.,^7^ Lee et al.,^10^ and Schlögl et al.^11^ The thyroid volume of each lobe was approximated by an ellipsoid using Eq. 4; then, the sum of each lobe was taken as the thyroid volume (*V*_ellipsoid_).

(4) $$\begin{array}{c} \begin{array}{c} V_{\text{ellipsoid}}\left[{\text{cm}}^{3}\right]={\left(\frac{\pi }{6}\times a_{L}\times b_{L}\times c_{L}\right)}_{\text{Leftlobe}}+{\left(\frac{\pi }{6}\times a_{R}\times b_{R}\times c_{R}\right)}_{\text{Rightlobe}} \end{array} \end{array}.$$

where *a* (cm) is a diameter and *b* (cm) is its orthogonal diameter. Each line passes through the centroid of the thyroid ROI on the cross‐sectional plane when the thyroid is approximated by an ellipsoid. *c* (cm) corresponds to the length of the major axis of the ellipsoid. The subscripts *L* and *R* indicate left and right, respectively. In this study, the isthmus was not considered in the volume calculation.

**Fig. 2:**
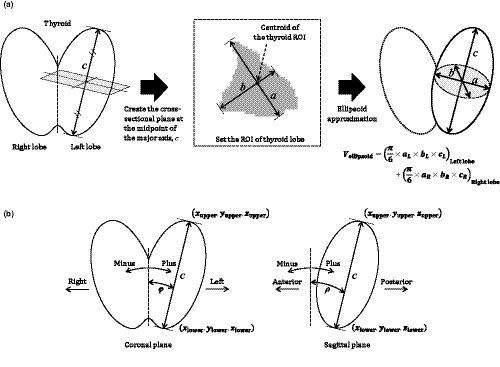
(a) Calculation method for thyroid volumes by the ellipsoid approximation method. (b) Calculation method for the length of the major axis, *c*, and the angles, φ, and ρ, in each patient's thyroid lobe.

The calculation flow is summarized in Fig. 2(a). First, the calculation for the major axis, *c*, will be described. We set the troid ROI at the top and bottom of each thyroid lobe on the CT image manually and calculated the centroid coordinates of each ROI: (*x*_upper_, *y*_upper_, *z*_upper_) and (*x*_lower_, *y*_lower_, *z*_lower_). *c* was calculated from these coordinates according to Equation 5. In addition, we obtained the angles, φ (formed by *c* and the coronal plane) and ρ (formed by *c* and the sagittal plane), as shown in Fig. 2(b), and Eqs. (6) and (7).

(5) $$c\left[\text{cm}\right]=\sqrt{{\left(x_{\text{upper}}‐x_{\text{lower}}\right)}^{2}+{\left(y_{\text{upper}}‐y_{\text{lower}}\right)}^{2}+{\left(z_{\text{upper}}‐z_{\text{lower}}\right)}^{2}}$$

(6) $$\varphi \left[\deg.\right]=artcan\left(\frac{y_{\text{upper}}‐y_{\text{lower}}}{z_{\text{upper}}‐z_{\text{lower}}}\right)$$

(7) $$\begin{array}{c} \begin{array}{c} \rho \left[\text{deg}.\right]=arctan\left(\frac{x_{\text{upper}}‐x_{\text{lower}}}{z_{\text{upper}}‐z_{\text{lower}}}\right) \end{array} \end{array}$$

**Fig. 3:**
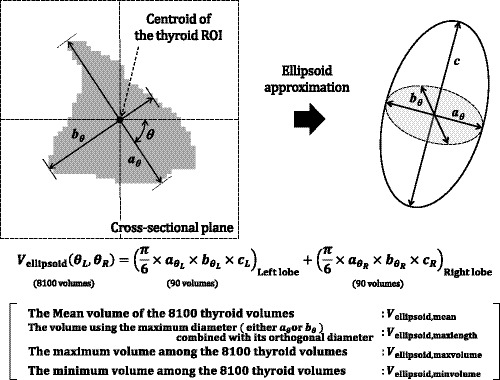
Measurement of arbitrary diameters and their corresponding orthogonal diameters (*a* and *b*) on the re‐sliced cross‐sectional plane at the midpoint of the major axis. Four approximate volumes (*V*_ellipsoid,mean_, *V*_ellipsoid,maxlength_, *V*_ellipsoid,maxvolume_, and *V*_ellipsoid,minvolume_) were obtained from *a* and *b*.

**Fig. 4:**
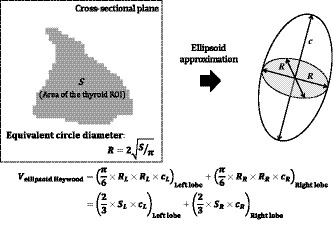
Ellipsoid approximation method for thyroid volumes using the equivalent circle diameter (Heywood diameter).

Subsequently, to calculate *a* and *b*, we created the cross‐sectional plane at the midpoint of the major axis, *c*. Furthe.more, according to the definition of the ellipsoid, the cross‐sectional plane is perpendicular to the two planes (containing *c*). In this study, we limited the plane for defining *a* and *b* to *the cross‐sectional plane at the midpoint of the major axis*. Hereinafter, the *cross‐sectional plane* refers to *the "re‐sliced" cross‐sectional plane at the midpoint of the major axis*. The thyroid ROI of each thyroid lobe was set manually for this *re‐sliced cross‐sectional plane* cropped to a 192 × 192 matrix (Figs. 2, 3, 4). As shown in Fig. 3, the centroid of the thyroid ROI in the.cross‐sectional plane was determined for each thyroid lobe. Furthermore, the combination of the diameter, *a*_θ_, and its orthogonal diameter, *b*_θ_, at angle θ were set to pass through the centroid of the thyroid ROI. The diameter of the thyroid in the cross‐sectional plane can be obtained innumerable, regardless of whether it passes through the centroid of thyroid ROI. However, in this study, we restricted the diameter to those that pass through the centroid to define a strict ellipsoid. If θ is rotated from 0° to 90°, a combination of diameters (*a*_θ_, *b*_θ_) can be obtained. Considering the size of the thyroid lobe, a combination of diameters was automatically obtained every 1° in each thyroid lobe (i.e., 90 combinations). Finally, a total of 8100 thyroid volumes per patient were acquired using 90 combinations of each thyroid lobe as shown in Eq. (8).

(8) $$\begin{array}{c} \begin{array}{c} V_{\text{ellipsoid}}\left(\theta_{L},\theta_{R}\right)\left[{\text{cm}}^{3}\right]={\left(\frac{\pi }{6}\times a_{\theta_{L}}\times b_{\theta_{L}}\times c_{L}\right)}_{\text{Leftlobe}}+{\left(\frac{\pi }{6}\times a_{\theta_{R}}\times b_{\theta_{R}}\times c_{R}\right)}_{\text{Rightlobe}} \end{array} \end{array}.$$

As a different concept from the one above, Heywood^13^ reported that the ellipsoid approximation method can be satisfactorily performed using an equivalent circle diameter (Heywood diameter) as the diameter of the cross‐sectional plane. In this study, we also verified the accuracy of the approximate volume derived from the equivalent circle diameter (Fig. 4). Assuming that the area of the thyroid ROI in the cross‐sectional plane is *S* (cm^2^), the equivalent circle diameter, *R* (cm), can be expressed as follows using *S*:

(9) $$\begin{array}{c} \begin{array}{c} R\left[\text{cm}\right]=2\sqrt{\frac{S}{\pi }} \end{array} \end{array}.$$

Eqs. (10 and 11) are obtained by substituting *R* into *a* and *b* in Eq. (8).

(10) $$\begin{array}{c} \begin{array}{c} V_{\text{ellipsoid,Heywood}}\left[{\text{cm}}^{3}\right]={\left(\frac{\pi }{6}\times R_{L}\times R_{L}\times c_{L}\right)}_{\text{Leftlobe}}+{\left(\frac{\pi }{6}\times R_{R}\times R_{R}\times c_{R}\right)}_{\text{Rightlobe}} \end{array} \end{array}.$$

(11) $$\begin{array}{c} \begin{array}{c} V_{\text{ellipsoid,Heywood}}\left[{\text{cm}}^{3}\right]={\left(\frac{2}{3}\times S_{L}\times c_{L}\right)}_{\text{Leftlobe}}+{\left(\frac{2}{3}\times S_{R}\times c_{R}\right)}_{\text{Rightlobe}} \end{array} \end{array}.$$

Among the thyroid volumes, *V*_ellipsoid_(*θ*_L_,*θ*_R_), obtainable by the methods described in Figs. 3, 4 and Eqs. ((8), (9), (10), (11)), five approxate volumes were compared with those determined three dimensionally from CT images. These volumes are as follows: (a) the mean volume of the 8100 thyroid volumes, (*V*_ellipsoid,mean_), (b) the volume using the maximum diameter (either *a*_θ_ or *b*_θ_) combined with its orthogonadiameter, (*V*_ellipsoid,maxlength_), (c) the maximum volume among the 8100 thyroid volumes, (*V*_ellipsoid,maxvolume_), (d) the minimum volume among the 8100 thyroid volumes, (*V*_ellipsoid,minvolume_), an(e) the thyroid volume determined with equivalent circle diameter (Heywood diameter), (*V*_ellipsoid,Heywood_). From these relationships, the accuracy and validity of the ellipsoid approximation method were examined, and the optimal method was identified.

### 2.4 Statistical analysis

We performed a simple regression analysis between the five approximate volumes and the three‐dimensionally extracted thyroid volume, that is, *V*_CT_, in the statistics software package, R (version 3.5.1 for Windows). The error rate between the five approximate volumes (*V*_ellipsoid_) and *V*_CT_ was obtained using Eq. (12).

(12) $$\begin{array}{c} \begin{array}{c} E_{\text{ellipsoid}}\left[\%\right]=100\times \frac{V_{\text{ellipsoid}}‐V_{\text{CT}}}{V_{\text{CT}}} \end{array} \end{array}.$$

Furthermore, we calculated a Lin's concordance correlation coefficient (CCC) according to Equation 13 to examine the consistency between *V*_CT_ and *V*_ellipsoid_ in each patient.^14^

(13) $$\begin{array}{c} \begin{array}{c} CCC=\frac{2\cdot \text{COV}\left(V_{\text{CT}},V_{\text{ellipsoid}}\right)}{\sigma_{V_{\text{CT}}}^{2}+\sigma_{V_{\text{ellipsoid}}}^{2}+{\left(\mu_{V_{\text{CT}}}‐\mu_{V_{\text{ellipsoid}}}\right)}^{2}}. \end{array} \end{array}$$

where COV (*V*_CT_, *V*_ellipsoid_) is the covariance between *V*_CT_ and *V*_ellipsoid_, $\sigma_{V_{\text{CT}}}^{2}$ and $\sigma_{V_{\text{ellipsoid}}}^{2}$ are the variances of *V*_CT_ and *V*_ellipsoid_, respectively, and $\mu_{V_{\text{CT}}}$ and $\mu_{V_{\text{ellipsoid}}}$ are the mean of *V*_CT_ and *V*_ellipsoid_, respectively.

Bride^15^ suggested the following scale to describe the correlation based on values of CCC; <0.90: Poor, 0.90–0.95: Moderate, 0.95–0.99: Substantial, >0.99: Almost perfect. Similar to the study of Lee et al.,^10^ we compared the CCC values for the five approximate volumes with McBride's sle.

## 3 Results

**Fig. 5:**
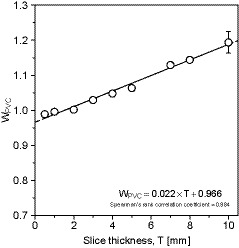
Relationship between the slice thickness, *T* and *W*_PVC_, the regression equation was *W*_PVC_ = 0.022 × *T* + 0.966. At 5 mm slice thickness, *W*_PVC_ was 1.077, which was about 8% overestimated over true volume.

First, when the linear regression equa.n was obtained from the relationship between the slice thickness, *T* and *W*_PVC_, the regression equation was *W*_PVC_ = 0.022 × *T* + 0.966 (Fig. 5). At 5‐mm slice thickness, *W*_PVC_ was 1.077, which was about 8% overestimated over true volume. Therefore, the measured volume obtained in the 5‐mm slice was divided by 1.077, and the partial volume‐corrected thyroid volume was used as the reference data (*V*_CT_).

**Fig. 6:**
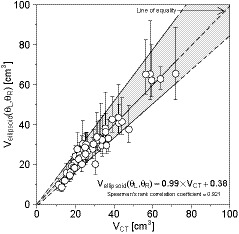
Relationship between *V*_CT_ and the 8100 thyroid volumes obtained by combining *a* and *b* of each thyroid lobe [*V*_ellipsoid_(*θ*_L_,*θ*_R_)]. The upper and lower end of the error bar represents the maximum and the minimum thyroid volume, respectively. The shaded area indicates an area surrounded by the regression lines of *V*_ellipsoid,maxvolume_ and *V*_ellipsoid,minvolume_ (Corresponding to Figs. 7(c) and 7(d), respectively). The variation in the approximate volumes tended to increase as the thyroid volume increased.

Table 1 shows the length of the major axis, *c*, and the angles, φ and ρ, in all 50 cases. The mean length of *c* on both thyroid lobes is approximately 6 cm. The angle, φ, is 8.30 ± 3.28° in the left lobe and −7.65 ± 3.14° in the right lobe. Similarly, the angle, ρ, is 0.19 ± 4.95° in the left lobe and 1.02 ± 5.41° in the right lobe. Figure 6 shows the relationship between *V*_CT_ and the 8100 thyroid volumes per patient obtained by systematically combining *a* and *b* of each thyroid lobe [*V*_ellipsoid_(*θ*_L_,*θ*_R_)]. The upper end of the error bar represents the maximum thyroid volume (*V*_ellipsoid,maxvolume_), and the lower end represents the minimum thyroid volume (*V*_ellipsoid,minvolume_). Thyroid volumes obtained by the ellipsoid approximation method varied by changing the combination of *a* and *b*. In particular, the variation in the approximated volume tended to increase as the thyroid volume increased (shaded area in Fig. 6).

**Table 1:** Length of the major axis, *c*, and the angles, φ and ρ, in all 50 cases.

Right lobe			Left lobe		
*c*_R_ (cm)	φ (deg.)	ρ (deg.)	*c*_L_ (cm)	φ (deg.)	ρ (deg.)
6.6 ± 1.2	‐7.65 ± 3.14	1.02 ± 5.41	6.2 ± 1.2	8.30 ± 3.28	0.19 ± 4.95

Data are expressed as mean ± 1 standard deviation (SD).

**Fig. 7:**
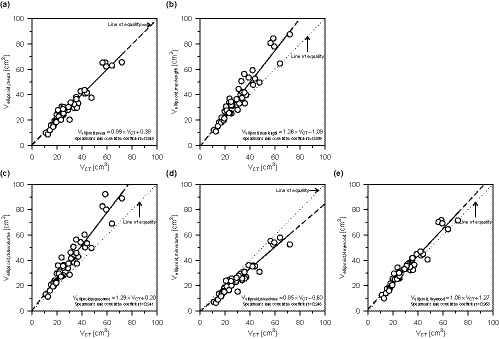
Relationship between *V*_CT_ and the five approximate volumes (*V*_ellipsoid,mean_, *V*_ellipsoid,maxlength_, *V*_ellipsoid,maxvolume_, *V*_ellipsoid,minvolume_, and *V*_ellipsoid,Heywood_). Although there was a strong correlation between *V*_CT_ and these approximate volumes, the slopes of the regression equation were different for each relationship (0.85–1.29). For this reason, CCC differed for each approximate volume (0.774–0.943).

**Fig. 8:**
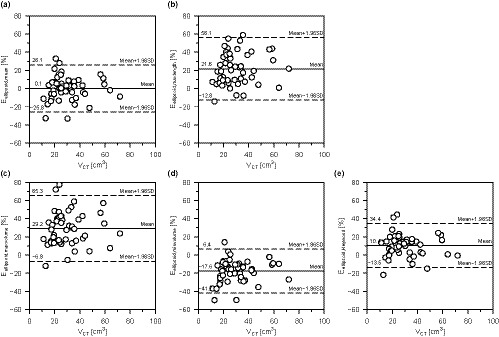
Error rate between *V*_CT_ and the five approximate volumes (*E*_ellipsoid,mean_, *E*_ellipsoid,maxlength_, *E*_ellipsoid,maxvolume_, *E*_ellipsoid,minvolume_, and *E*_ellipsoid,Heywood_). The mean error rate was the highest between *V*_ellipsoid,maxvolume_ and *V*_CT_ (29.2%), and the lowest between *V*_ellipsoid,mean_ and *V*_CT_ (0.1%).

Table 2 shows the thyroid volumes obtained by three‐dimensional volumetry (*V*_CT_) and the ellipsoid approximation method (*V*_ellipsoid,mean_, *V*_ellipsoid,maxlength_, *V*_ellipsoid,maxvolume_, *V*_ellipsoid,minvolume_, and *V*_ellipsoid,Heywood_). Each relationship is shown in Figs. 7(a)–7(e), and the error rates from *V*_CT_ and the ellipsoid approximation method (*E*_ellipsoid,mean_, *E*_ellipsoid,maxlength_, *E*_ellipsoid,maxvolume_, *E*_ellipsoid,minvolume_, and *E*_ellipsoid,Heywood_) are shown in Figs. 8(a)–8(e). *V*_CT_ as the reference volume was widely distributed between 11.2 and 71.9 cm^3^. Table 3 lists the values of the correlation coefficient, CCC, and McBride's scale for the thyroid volumes calculated by the ellipsoid approximation method when compared to *V*_CT_. All correlations are strong with correlation coefficients of 0.900 or higher. The mean error rate was the highest between *V*_ellipsoid,maxvolume_ and *V*_CT_ (29.2%), and the lowest between *V*_ellipsoid,mean_ and *V*_CT_ (0.1%). CCC was used to assess the consistency of each relationship; the highest was for *V*_ellipsoid,mean_ (0.943; moderate) and the lowest was for *V*_ellipsoid,maxvolume_ (0.774; poor).

**Table 2:** Thyroid volumes calculated by three‐dimensional volumetry (*V*_CT_) and the ellipsoid approximation method (*V*_ellipsoid,mean_, *V*_ellipsoid,maxlength_, *V*_ellipsoid,maxvolume_, *V*_ellipsoid,minvolume_, and *V*_ellipsoid,Heywood_).

*V*_CT_ (cm^3^)	*V*_ellipsoid,mean_ (cm^3^)	*V*_ellipsoid,maxlength_ (cm^3^)	*V*_ellipsoid,maxvolume_ (cm^3^)	*V*_ellipsoid,minvolume_ (cm^3^)	*V*_ellipsoid,Heywood_ (cm^3)^
30.1 ± 13.8	30.2 ± 14.1	37.0 ± 18.3	39.1 ± 18.7	25.0 ± 12.3	33.2 ± 15.1

Data are expressed as mean ± 1 standard deviation (SD).

**Table 3:** Correlation coefficient, CCC, and McBride's scale between *V*_CT_ and each approximate volume. CCC for assessing the consistency of each relationship was above 0.900 for *V*_ellipsoid,mean_ and *V*_ellipsoid,Heywood._

	*V*_ellipsoid,mean_	*V*_ellipsoid,maxlength_	*V*_ellipsoid,maxvolume_	*V*_ellipsoid,minvolume_	*V*_ellipsoid,Heywood_
Correlation coefficient*	0.943	0.939	0.941	0.945	0.963
Lin’s CCC	0.943	0.821	0.774	0.866	0.924
McBride's scale	Moderate	Poor	Poor	Poor	Moderate

* Spearman's rank correlation coefficient.

## 4 Discussion

As shown in Fig. 5, the true volume, *V*_true_ and the measured volume, *V*_measured_ are equal when the slice thickness is 2 mm or less; however, the difference between both volumes increases due to the influence of the partial volume effect when the slice thickness is 2 mm or more. Hence, partial volume correction is recommended when performing thyroid volumetry using CT images with slice thickness thicker than 2 mm.

Thyroid volumes obtained by the ellipsoid approximation method varied by changing the combination of *a* and *b* as shown in Fig. 6. In particular, the variation in the approximated volumes tended to increase as the thyroid volume increased. Generally, when performing thyroid volumetry on an ultrasound image, the length of the cross‐sectional plane is subjectively determined by the operator. Thyroid volumetry using the ellipsoid approximation method that requires the lengths, *a* and *b*, is prone to error. According to Figs. 7 and 8, and Table 3, although there was a correlation between *V*_CT_ and the ellipsoid approximation volumes, CCC differed depending on the approximate volume (0.774–0.943). *V*_ellipsoid,maxlength_, *V*_ellipsoid,maxvolume_, and *V*_ellipsoid,Heywood_ were overestimated by 21.6%, 29.2%, and 10.4% respectively, and *V*_ellipsoid,minvolume_ was underestimated by 17.6%. Schlögl et al.^11^ reported that the approximate volume was 11% larger than the actual volume. Our study supported their findings because they used the maximum diameter to approximate the ellipsoid. In radioiodine therapy for Graves' disease, any error in the measurement of the thyroid volume directly affects the absorbed dose by the thyroid, and thereby the therapeutic effect, as shown in Eq. (1). The success rate of internal radioiodine therapy in Graves' disease has been reported by Peters et al.^16^ to depend on the thyroid absorbed dose and thyroid weight. By applying these errors to Marinelli's formula [Eq. (1)] and calculating backwards with the administered radioactivity, *A*, as a constant, the thyroid absorbed doses, *D*, are reduced by 17.8%, 22.6%, and 9.5% when *V*_ellipsoid,maxlength_, *V*_ellipsoid,maxvolume_, and *V*_ellipsoid,Heywood_ are used as thyroid volumes, respectively. Similarly, when *V*_ellipsoid,minvolume_ is used, the thyroid absorbed dose is 21.4% in excess of the reference. In Marinelli's formula, radioiodine uptake fraction and effective iodine half‐life are also used to determine the administered radioactivity, so these parameters other than thyroid volume can also be an error factor. Because our study focuses on thyroid volume variations, another study is needed to investigate variations due to other parameters. The SNMMI practice guideline for therapy of thyroid disease with iodine‐131^17^ recommend 3–8 MBq/g as the volume‐adjusted dose of administered radioactivity. This method is simple and is practiced in many facilities. If we determine the administered radioactivity using a volume‐adjusted dose method, only thyroid volume is an error factor of the administered radioactivity. From the above points, we need to measure the thyroid volume more accurately. In addition, according to this SNMMI practice guideline, iatrogenic hypothyroidism by overdose is acceptable, but considering the time and effort of oral levothyroxine, we should achieve the euthyroid state as much as possible. On the contrary, underdose can result in retreatment being required, and should be avoided. For these reasons, accurate thyroid volume measurements could prevent overdose or underdose and determine the appropriate administered radioactivity. Conversely, Leslie et al.^18^ reported that a volume‐adjusted dose (2.96 or 4.44 MBq/g) of administered radioactivity has no advantage over a completely fixed dose (235 or 350 MBq). There is an ongoing discussion on whether to determine the administered radioactivity using volume‐adjusted dose or completely fixed dose.^3^

*V*_ellipsoid,mean_ can be obtained by automatically measuring the volume of the thyroid using a combination of *a* and *b* and then averaging these multiple volumes (8100 volumes per patient in this study); thus, the mean error rate with *V*_CT_ could be reduced to 0.1% because of error cancellation due to averaging. In contrast, *V*_ellipsoid,Heywood_ was in good agreement with *V*_CT_ according to the results of single regression analyses and the resultant CCC values; the mean error rate with *V*_CT_ was 10.4%. There are no reports of past studies in which the thyroid volumes were calculated by the ellipsoid approximation method using the equivalent circle diameter. Therefore, this result represents a new finding. For *V*_ellipsoid,Heywood_, it is not necessary to consider the variation due to *a* and *b* because this approximated thyroid volume is calculated from the equivalent circle diameter derived from the thyroid ROI on the cross‐sectional plane. Therefore, if the ROI can be set accurately in the thyroid region, the accuracy and robustness of the ellipsoid approximation method should be higher than that for the conventional ellipsoid diameters.

We also considered the length of the major axis, *c*. The angle between *c* and the coronal plane was approximately 8°, and the angle between *c* and the sagittal plane was approximately 1°, for all cases. At these angles, the error between the projected length of *c* on the coronal or sagittal plane and the actual length of *c* was approximately 1%. Lee et al.^10^ measured the length of the major axis on the coronal or sagittal planes and calculated the thyroid volume by the ellipsoid approximation method. Our study supported the validity of measuring the axial length from the coronal or sagittal planes described in their method. The length of the major axis could be measured accurately and easily if we use CT images. An arbitrary cross‐sectional image can be obtained using ultrasonography, although this route is prone to subjectivity in measurement. In recent years, it has become possible to acquire volume images with less distortion using three‐dimensional ultrasound images. If the knowledge of this study is applied to ultrasound images, thyroid volumetry could be acquired more easily and accurately in the future.

For each volume determined in this study, the error due to the ellipsoid approximation method was in the range, 0–50%, and in some cases the error exceeded 50%. Although it is possible to easily obtain an approximate thyroid volume by the ellipsoid approximation method, the limitation is the approximation itself. Even *V*_ellipsoid,mean_ and *V*_ellipsoid,Heywood_ contains non‐negligible errors due to the approximation. Therefore, we conclude that thyroid volumetry using the ellipsoid approximation cannot be a viable alternative to three‐dimensional thyroid volumetry in radioiodine therapy; it is necessary to perform three‐dimensional thyroid volumetry using CT or ultrasound images for therapeutic efficacy.

In this study, we set *the re‐sliced cross‐sectional plane at the midpoint of the major axis* according to the definition of the ellipsoid, and obtained the cross‐sectional diameters, *a* and *b*, on this plane. In clinical practice, the cross‐sectional diameter of the thyroid is often obtained from the *trans‐axial plane of the body* (*cranial to caudal axis*) using CT or ultrasonography. That is, *the re‐sliced cross‐sectional plane at the midpoint of the major axis* in the ellipsoid is inclined with *the trans‐axial plane of a body* (Table 1). In the 50 cases of this study, even if *the trans‐axial plane of a body at the midpoint of the major axis* was used in place of *the re‐sliced cross‐sectional plane at the midpoint of the major axis*, the difference between the cross‐sectional areas of the two planes was 1% or less. Therefore, the inclination of the cross‐sectional plane cannot be a large variation factor. However, it can be concluded that the cross‐sectional diameters (*a*, *b*) or area (*S*) are the main parameters affecting the ellipsoid volume.

Patients with Graves' disease tend to have low CT values in the thyroid. There were some cases where it was difficult to extract thyroid regions in this study. If the CT value of the thyroid is low, the accuracy of the extraction becomes a problem. Although Shu et al. and Lee et al. reported good agreement for the results of three‐dimensional thyroid volumetry using contrast‐enhanced CT images with the actual thyroid volume,^9^, ^10^ one of the limitations of our study is that the measurement accuracy of three‐dimensional volumetry was not examined. Contrast‐enhanced CT scans will improve thyroid visibility and segmentation, but they will delay radioiodine therapy for weeks or months because the contrast media contains iodine.^3^, ^4^ In addition, we need to consider the inter‐ and intraoperator error of the manually extracted‐thyroid ROI. Veres et al.^12^ reported that the interoperator error was about 6% and the intraoperator error was about 3% when the thyroid was manually segmented from CT images. Similarly, Nygaard et al.^19^ reported that the interobserver error was 11% and the intra‐operator errors were 4 and 6% for the two operators, respectively. With such manual segmentation, there may be a few percent of inter‐ and intra‐operator errors.

In contrast, visibility of the thyroid is improved in ultrasound or magnetic resonance (MR) images. Therefore, it would be easier to extract the region, although determining the cross‐sectional plane of the thyroid lobe could be difficult. Also, the ultrasound examination is relatively low‐cost compared to CT or MR examination. Nygaard et al.^19^ performed thyroid volumetry from cross‐sectional imaging by CT and ultrasound. They reported that a significant correlation was found between the thyroid volume measured by CT and ultrasound (Spearman's rank correlation coefficient = 0.945), although the thyroid volume measured by ultrasound was 17% smaller than the thyroid volume measured by CT. They also reported that the maximum variation between the thyroid volume calculated from the cross‐sectional image and the thyroid volume calculated from the ellipsoid approximation was 129% when using ultrasound images (the median variation was 23%). Similarly to our study, their study measured thyroid volume with a slice thickness of 5 mm for both CT and ultrasound images, but it may include the influence of the partial volume effect because they did not apply the partial volume correction. If a thin cross‐sectional image similar to CT can be used for ultrasound images, the results of this study could be reflected in ultrasound images. There are also some reports of thyroid volumetry using MR. Huysmans et al.^20^ reported that thyroid volumetry using MR have very good reproducibility. In addition, Isselt et al.^21^ conducted a study using the volume data obtained by MR as the gold standard. MR images provide excellent delineation of the thyroid from the surrounding tissues. They also report that MR volumetry is well standardized and validated, and its reproducibility (with errors of 1–2%) is very good. On the other hand, they also mention “the limited availability and capacity, as well as the relatively high cost, restrain the clinical application of MRI for thyroid volume measurements in patients with Graves' disease.” As a result, it seems impractical to perform an MR examination solely for thyroid volumetry. In our hospital, we measure thyroid volumes using CT images. Therefore, ultrasound or MR images were not available as part of a retrospective study. Further studies are needed to verify the accuracy of our five ellipsoid‐approximation‐method volumes using ultrasound or MR images to extract reference volume data.

As reported in previous studies, there is no gold standard for thyroid volumetry. Even if we measure the volume of the specimen, we cannot obtain the true volume owing to surgical manipulation and bleeding.^8^, ^10^, ^19^, ^20^ When we calculate the thyroid volume by ellipsoid approximation, it is intuitively understandable that the approximated volume varies depending on the measured length if the cross‐sectional shape is complicated. Because there is no gold standard for thyroid volumetry, it is not possible to compare the true volume with the measured volume. However, the relative variability of the ellipsoid approximation volume due to the measured length can be evaluated. Therefore, we find the research value of this study in that we obtained the relative variation of thyroid volume due to the measured length.

## 5 Conclusion

We determined the variation in the approximated thyroid volumes determined by the ellipsoid approximation method using CT images; thyroid volumes varied depending on the diameter or area of the cross‐sectional plane. The mean error rate of *V*_ellipsoid,mean_ with *V*_CT_ was almost zero (0.1%). Furthermore, we found that the ellipsoid approximation method using the equivalent circle diameter can determine thyroid volumes in good agreement with those extracted manually by three‐dimensional volumetry. The mean error rate of this method, *V*_ellipsoid,Heywood_ with *V*_CT_ could be reduced to 10.4%. However, thyroid volumetry by the ellipsoid approximation method included an error in the range 0–50% and in some cases the error exceeded 50%. Consequently, we recommend three‐dimensional thyroid volumetry if measurement accuracy is required.

## 6 Ethics approval and consent to participate

All procedures performed in studies involving human participants were in accordance with the ethical standards of the Ethical Review Committee of Nagoya University Hospital and with the 1964 Helsinki Declaration and its later amendments or comparable ethical standards. This study was approved by the Ethical Review Committee of Nagoya University Hospital (authorization no. 2018‐0179). Since this study analyzed clinical data retrospectively, we did not obtain informed consent directly from each patient. Instead of direct informed consent, we published the research contents on the university web page and provided an opt‐out opportunity.

## Conflicts of Interest

The authors declare that they have no conflict of interest.
